# Cloning and Characterization of the Human Trefoil Factor 3 Gene Promoter

**DOI:** 10.1371/journal.pone.0095562

**Published:** 2014-04-17

**Authors:** Yong Sun, Liangxi Wang, Yifang Zhou, Xuefei Mao, Xiangdong Deng

**Affiliations:** 1 Department of Burn Surgery, Huaihai Hospital affiliated to Xuzhou Medical College, Xuzhou, Jiangsu Province, China; 2 Department of Burn Surgery, No. 97 Hospital of PLA, Xuzhou, Jiangsu Province, China; University of Massachusetts Medical, United States of America

## Abstract

Human trefoil factor 3 (hTFF3) is a small-molecule peptide with potential medicinal value. Its main pharmacological function is to alleviate gastrointestinal mucosal injuries caused by various factors and promote the repair of damaged mucosa. However, how its transcription is regulated is not yet known. The aim of this study was to clone the *hTFF3* gene promoter region, identify the core promoter and any transcription factors that bind to the promoter, and begin to clarify the regulation of its expression. The 5′ flanking sequence of the *hTFF3* gene was cloned from human whole blood genomic DNA by PCR. Truncated promoter fragments with different were cloned and inserted into the pGL3-Basic vector to determine the position of the core *hTFF3* promoter. Transcription element maintaining basic transcriptional activity was assessed by mutation techniques. Protein-DNA interactions were analyzed by chromatin immunoprecipitation (ChIP). RNA interference and gene over-expression were performed to assay the effect of transcription factor on the *hTFF3* expression. The results showed that approximately 1,826 bp of the fragment upstream of *hTFF3* was successfully amplified, and its core promoter region was determined to be from −300 bp to −280 bp through analysis of truncated mutants. Mutation analysis confirmed that the sequence required to maintain basic transcriptional activity was accurately positioned from −300 bp to −296 bp. Bioinformatic analysis indicated that this area contained a Sp1 binding site. Sp1 binding to the *hTFF3* promoter was confirmed by ChIP experiments. Sp1 over-expression and interference experiments showed that Sp1 enhanced the transcriptional activity of the *hTFF3* promoter and increased *hTFF3* expression. This study demonstrated that Sp1 plays an important role in maintaining the transcription of *hTFF3*.

## Introduction

Human trefoil factor 3 (hTFF3) is a small polypeptide specifically secreted by the intestinal goblet cells [1]. There is a special core structural domain within the 59-amino acid sequence, in which six cysteines are connected to each other by disulfides bridges in a specific order, forming a three-cyclic structure, whereby the whole peptide chain is bent and folded and shaped like a “clover.” This is the so called trefoil factor. Since its discovery, hTFF3 has been a target of study for many researchers. A large number of studies showed that hTFF3 occupies an important niche in the self-protection and repair mechanisms of the intestinal mucosa [2], [3]. Regulation of hTFF3 is complex and precise, and, although a variety of substances are known to be involved in the regulation of hTFF3 expression [4], [5], [6], the basal regulation of *hTFF3* transcription is still unclear. Therefore, in this study, we aimed to clarify the regulation of *hTFF3* transcription. An approximately 1,826-bp fragment upstream of *hTFF3* was successfully amplified using genomic DNA extracted from whole blood, and its core promoter region was determined to be located from −300 bp to −280 bp through truncation analysis. Mutational analysis confirmed that the minimum sequence required for maintaining basic transcriptional activity was accurately positioned from −300 bp to −296 bp. Bioinformatic analysis confirmed that this area contained a Sp1 binding site. ChIP experiments confirmed that Sp1 binds to the *hTFF3* promoter. Sp1 over-expression and interference experiments showed that Sp1 enhanced the activity of the *hTFF3* promoter and increased *hTFF3* expression.

## Materials and Methods

### Ethics Statement

The study was approved by the ethics committee of No. 97 Hospital of PLA, and written informed consent was obtained from participants.

### Cell Culture

Both human embryonic kidney (HEK) 293 cells and LS-174T colon cancer cells were purchased from ATCC (Manassas, VA, USA). Cells were cultured in DMEM containing 10% fetal bovine serum, 100 U/mL penicillin, and 100 U/mL streptomycin, at 37°C and 5% CO_2_. The medium was changed every other day, and the cells were passaged every 3–4 days at a ratio of 1∶3. Properly shaped cells were selected for experimental research.

### Genomic DNA Isolation and Cloning of the *hTFF3* Promoter

Genomic DNA was extracted from human blood using a genomic DNA extraction kit (Promega, Madison, WI, USA). Primers were designed based on the sequence of the 5′ untranslated region of *hTFF3* (GenBank accession No. AB038162.1). The isolated genomic DNA was used as the template to amplify the *hTFF3* promoter region. KpnI and HindIII restriction sites were introduced into the forward and reverse primers, respectively. The PCR product was digested with KpnI and HindIII, and then ligated into pGL3-Basic, a plasmid that contains firefly luciferase, but no promoter. Positive clones were selected and sequenced. This plasmid was then used as the template for the amplification of truncated and mutated promoters by PCR. These PCR products were also digested with KpnI and HindIII, and then sequenced. AliBaba 2.1 was used to detect binding sites for transcription factors and to ensure new binding sites were not introduced by the mutations. All primers used are shown in Tables 1 and 2.

**Table 1:** Primers used for cloning the hTFF3 promoter.

Plasmids	Primer	Sequence 5′→3′
pGL3-1826	sense-1826	TATA**GGTACC** GGCAGGCAACGCTCTTTC
pGL3-1519	sense −1519	TATA**GGTACC** CCAAGTCCACCCTTCAGA
pGL3-1070	sense −1070	TATA**GGTACC** GCCTGTAATCCTGACACTTT
pGL3-700	sense −700	TATA**GGTACC**AAGTGTTCACAATAGATCAAT
pGL3-500	sense −500	TATA**GGTACC**GTTGTGAGAGAGCCGCAGGGT
pGL3-300	sense −300	TATA**GGTACC**TAGGAGGGCAATTGACACAC
pGL3-200	sense −200	TATA**GGTACC**TGACCTCTCCCCTTTGGGAG
pGL3-100	sense −100	TATA**GGTACC** AGCAAACAATCCAGAGCAGC
pGL3-280	sense −280	TATA**GGTACC** ATCCGCTCCCCAGTAGAGGA
pGL3-260	sense −260	TATA**GGTACC** CCCGGAACCAGAACTGGAAT
pGL3-240	sense −240	TATA**GGTACC** CCGCCCTTACCGCTTGCTGC
pGL3-220	sense −220	TATA**GGTACC** CAAAACAGTGGGGGCTGAAC
Commom	antisense+19	TTTA**AAGCTT**AGAGCGCTCTGGCAGCCAT

Restriction endonuclease sequences are shown in bold.

**Table 2:** Primers used for mutagenesis at the hTFF3 promoter.

Plasmids	Primer	Sequence 5′→3′
		–300 TAGGAGGGCAATTGACACAC −280
Mut-1	sense	TATAGGTACCGCTATGGGCAATTGACACACATC
Mut-2	sense	TATAGGTACCTAGGATACTGATTGACACACATCCGCTC
Mut-3	sense	TATAGGTACCTAGGAGGGCATACATCACACATCCGCTCCCCAG
Mut-4	sense	TATAGGTACCTAGGAGGGCAATTGAAGTCAATCCGCTCCCCAGTAGAG
Commom	antisense	TTTAAAGCTTAGAGCGCTCTGGCAGCCAT

Mutations were in the boxes.

### Construction of the Sp1 Expression Vector

The total RNA was extracted from HEK293 cells and reverse transcribed to obtain cDNA. Primer 5.0 software was used to design the following primers to amplify the *Sp1* gene sequence (GenBank accession No. NM_138473): forward, 5′-TAGAATTCATGAGCGACCAAGATCACTC-3′, and reverse, 5′-TACTCGAGTCAGAAGCCATTGCCACTGA-3′. PfuUltra DNA polymerase (Stratagene, La Jolla, CA, USA) was used to amplify the *Sp1* gene by PCR. The PCR products were digested with EcoRI and XhoI and ligated into pCDNA3.1 (+), which was also digested with EcoRI and XhoI. The resulting construct was then sequenced.

### Double-stranded Small Interfering RNA (siRNA)

Control siRNA and human SP1-specific siRNA were purchased from Santa Cruz Biotechnology (Santa Cruz, CA, USA), and were used to silence the expression of endogenous Sp1 by RNA interference according to the manufacturer’s instructions.

### Transfection and Luciferase Assays

HEK293 cells and LS174T cells in logarithmic growth phase were harvested and seeded in a 96-well plate. When the confluency reached 80%, the cells were transfected using the jetPEI kit (Polyplus-Transfection, France). The ratio of jetPEI to plasmid was 2 µL:1 µg. The transfected plasmids included pGL3-hTFF3, pGL3-Basic, and pGL3-control. The amount of transfected plasmid was 100 ng/well, and each plasmid was added to three wells. Each well was also transfected with 3 ng of pRL-TK plasmid as an internal reference. Six hours later, the medium was changed. Cells were screened after 48 h. Mithramycin A inhibits the binding of Sp1 to DNA. Cells were transfected with the reporter plasmid for 24 h, mithramycin A was added, and the cells were cultured for an additional 24 h. For Sp1 interference and over- expression experiments, 100 ng of luciferase reporter plasmid, 3 ng of pRL-TK, and the Sp1 interference or over-expression plasmid were co-transfected into cells and cultured for 48 h. Luciferase activity was measured using the Dual Reporter Assay System (Promega). The relative luciferase activity was calculated as the ratio of firefly luciferase to Renilla luciferase. At least three independent experiments were performed under similar experimental conditions.

### Chromatin Immunoprecipitation (ChIP) Assay

ChIP assays were performed using the ChIP-IT kit (Active Motif, USA) according to the manufacturer’s instructions. HEK293 cells or LS174T cells were conventionally cultured to approximately 70%–80% confluence, the medium was decanted, and then medium containing 1% formaldehyde was added to fix the cells. Cells were washed with cold PBS, and then glycine was added to terminate fixation, and the cells were washed again with cold PBS. After the cells were scaped from the culture plated, the cold cell lysate was centrifuged to collect the nuclei. The pelleted nuclei were resuspended, and chromatin was cleaved into approximately 500-bp fragments by ultrasonication. The cleaved chromatin was treated with RNase and proteinase K, and extent of cleavage was assessed by 1% agarose gel electrophoresis. Protein G beads were used to remove the non-specific antibodies from the chromatin preparation, Then, an anti-Sp1 antibody, a positive control RNA pol II antibody, and a negative control IgG antibody were added. Cells were incubated overnight at 4°C. Protein G beads were added to the solution containing the antibody/chromatin complexes, which were then washed and eluted, de-cross-linked, and the co-precipitated DNA was purified. Finally, the co-precipitated DNA was used as a template in a PCR amplification using the following primers: forward, 5′-CTGAGATGGAACGGACACC-3′ and reverse, 5′-TAAGGGCGGATTCCAGTT-3′.

### Quantitative Real-time RT-PCR

Total RNA was extracted from cells using TRIzol Reagent (Invitrogen, Carlsbad, CA, USA) according to the manufacturer’s instructions, and then the RNA was reverse transcribed using the PrimeScript RT Master Mix Perfect Real Time kit (TaKaRa, Dalian, China) to obtain the cDNA. Using the cDNA as the template, a real-time PCR assay was performed using the following two pairs of primers: hTFF3 forward, 5′-CCAAGGACAGGGTGGACTG-3′ and hTFF3 reverse, 5′-AAGGTGCATTCTGCTTCCTG-3′, and Sp1 forward, 5′-ACGCTTCACACGTTCGGATGAG-3′ and Sp1 reverse, 5′-TGACAGGTGGTCACTCCTCATG-3′. The 20 µL real-time PCR reaction included 0.5 µL of cDNA template, 0.25 µL of Primer F, 0.25 µL of Primer R, 10 µL of RNase-free dH_2_O, and 8 µL of 2.5× RealMasterMix (SYBR Green I). The reaction conditions included a pre-denaturation step at 95°C for 10 s, and 40 cycles of 95°C for 15 s and 60°C for 60 s. After the reaction, the data were subjected to statistical analysis.

### Western Blotting

The cells were collected, cracked, the protein concentration was quantified using the BCA Protein Assay Kit (Pierce, USA). Total protein (40 µg) was loaded in each lane and separated by 15% SDS-PAGE. After electrophoresis, the proteins were transferred to a nitrocellulose membrane, and probed with mouse anti-human polyclonal antibodies against hTFF3 and Sp1, and a monoclonal antibody against β-actin (Abcam, Cambridge, MA, USA), and then incubated overnight at 4°C. A horseradish peroxidase-labeled anti-mouse IgG was added and incubated at room temperature for 2 h with shaking. Chemiluminescence signals were quantified using an ECL imager, and analyzed using Quantity One software (Bio-Rad, Hercules, CA, USA).

### Statistical Analysis

All statistical analyses were performed using SPSS software (version 16.0). Data are presented as the mean ± SD. Results were analyzed using unpaired t-tests, and P values less than 0.05 were considered significant.

## Results

### Construction and Analysis of the Recombinant *hTFF3* Promoter Plasmid

**Figure 1:**
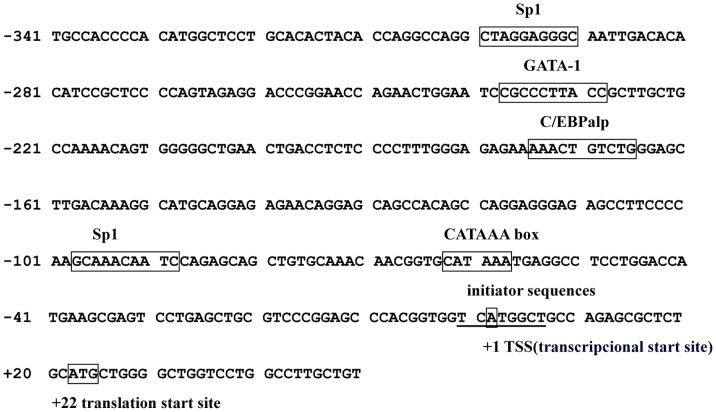
Nucleotide sequence of the *hTFF3* gene core promoter region. The numbering of the sequence is relative to the TSS. Putative binding sites for the transcriptional factors are boxed and labeled above.

**Figure 2:**
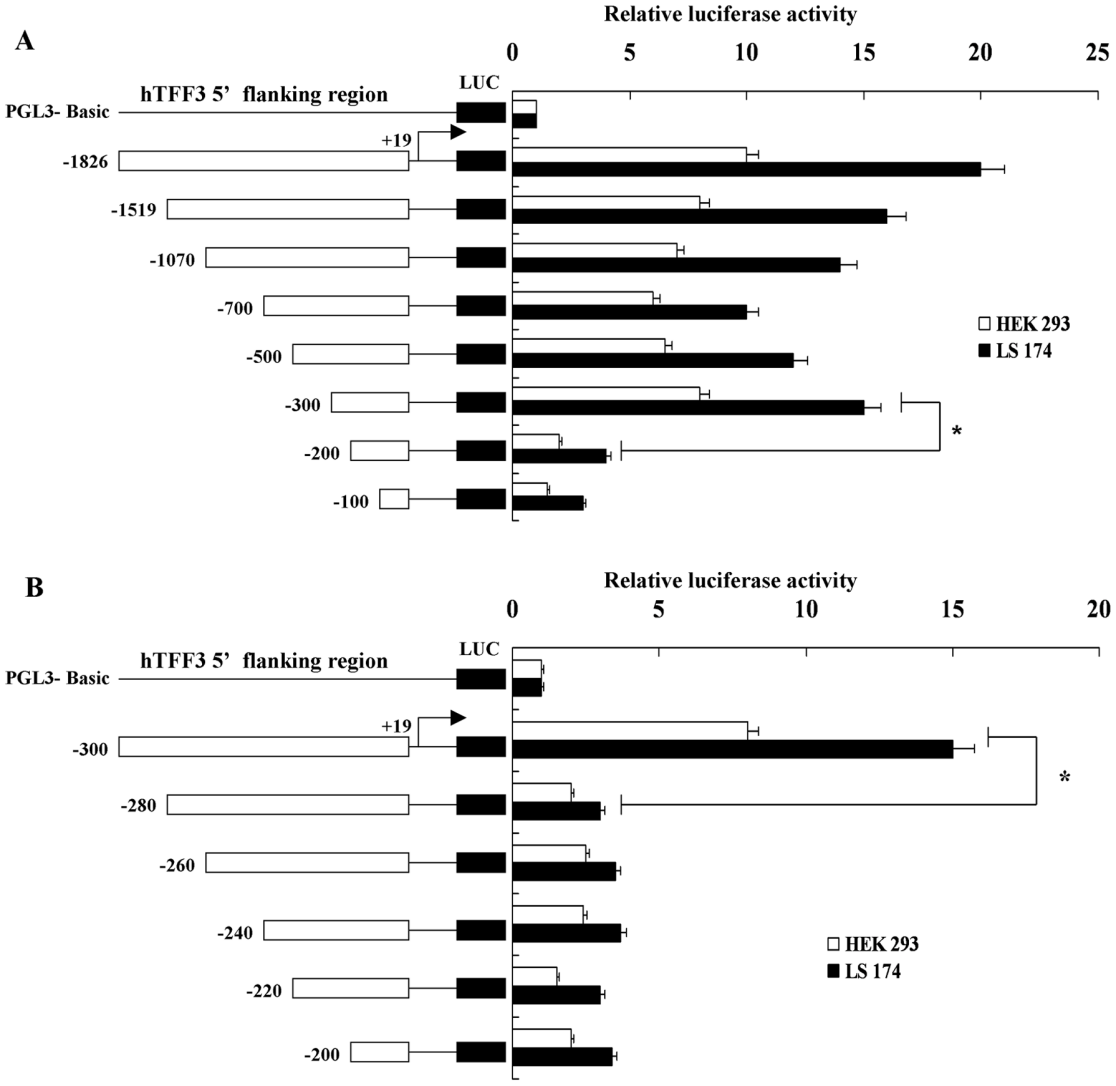
Analysis of *hTFF3* promoter activity. *hTFF3* promoters with different lengths were transfected into HEK 293 and LS-174T cells. The relative luciferase activity was calculated as the ratio of firefly luciferase to Renilla luciferase. At least three independent experiments were performed under similar experimental conditions. (A) the *hTFF3* promoter (−1,826 bp to −100 bp); B the *hTFF3* promoter (−300 bp to −200 bp). Data are presented as the mean ± SD (*P<0.05 *vs*. PGL3-200).

DNA sequences with different lengths at the upstream of the *hTFF3* gene were successfully amplified using genomic DNA extracted from normal human peripheral blood as the template (Table 1) (Fig.1). These sequences were cloned into the firefly luciferase plasmid pGL3-Basic. The recombinant plasmid was digested with KpnI and HindIII and sequenced, and the results showed that the sequence was identical to the sequence in GenBank (AB038162.1). The above plasmids and the internal reference plasmid were transfected into HEK 293 cells and LS-174T cells. After 48 h, relative luciferase activity was detected using a dual luciferase reporter gene system. As shown in Fig. 2A, the activities of all the luciferase reporter plasmids with different lengths of promoter were significantly higher in LS-174T cells than in HEK293 cells. The activities of the truncated vectors pGL3-1826–pGL3-300 were all higher than the negative control pGL3-basic; the activity of pGL3-1826 was 20 times higher, and the activity of pGL3-300 was 15 times higher. The activities of pGL3-200 and pGL3-100 were significantly lower (3–4 times) than the negative control. Therefore, the size of the promoter insert was further shortened, and four additional truncated vectors were constructed with 5′ ends starting from −300 bp to −200 bp, as shown in Fig. 2B. The activity of pGL3-300 was higher than the negative control; however, pGL3-280 through pGL3-220 had lower activities.

### Down-regulation of the Basal Transcriptional Activity of the *hTFF3* Promoter Upon Truncation from −300 bp to −296 bp

**Figure 3:**
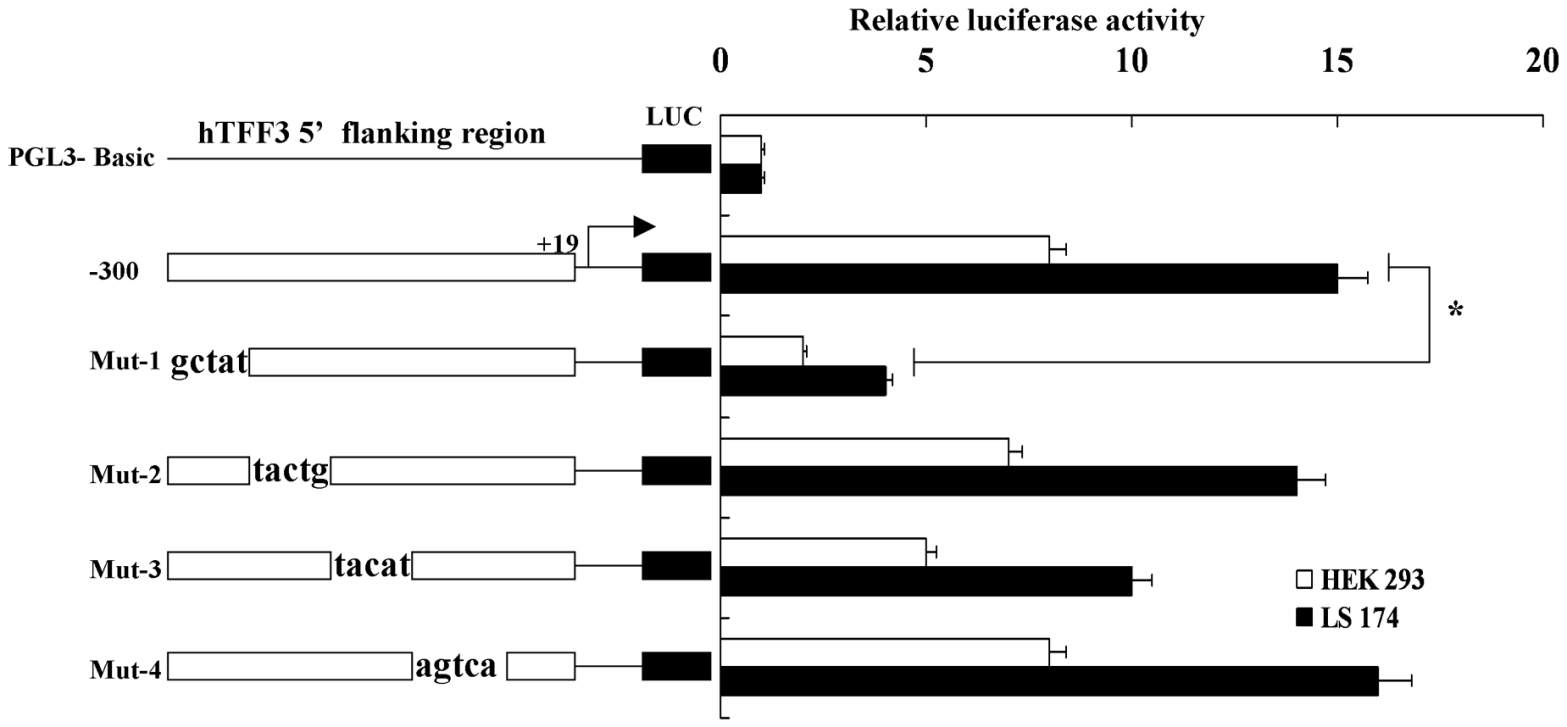
Mutation analysis of the *hTFF3* promoter. Four consecutive *hTFF3* promoter mutants were transfected into HEK 293 and LS-174T cells. The relative luciferase activity is presented as the ratio of firefly luciferase to Renilla luciferase. The results shown here were from three independent experiments. Data are presented as the mean ± SD (*P<0.05 *vs*. PGL3-300).

The region from −300 bp to −280 bp is essential for the basal transcriptional activity of the *hTFF3* promoter; therefore, we constructed four consecutive mutants focused on this 20-bp region (Table 2). As shown in Fig. 3, Mut-2, Mut-3, and Mut-4 had activities that were close to the activity of pGL3-300. However, the activity of Mut-1 was significantly decreased. Then, AliBaba 2.1 was used to search for transcription factor binding sites within the *hTFF3* promoter using the TRANSFAC database. There was exactly one SP1 binding site (−301 bp–−293 bp) in the Mut-1 region.

### Verification of the Interaction between Sp1 and the *hTFF3* Promoter

**Figure 4:**
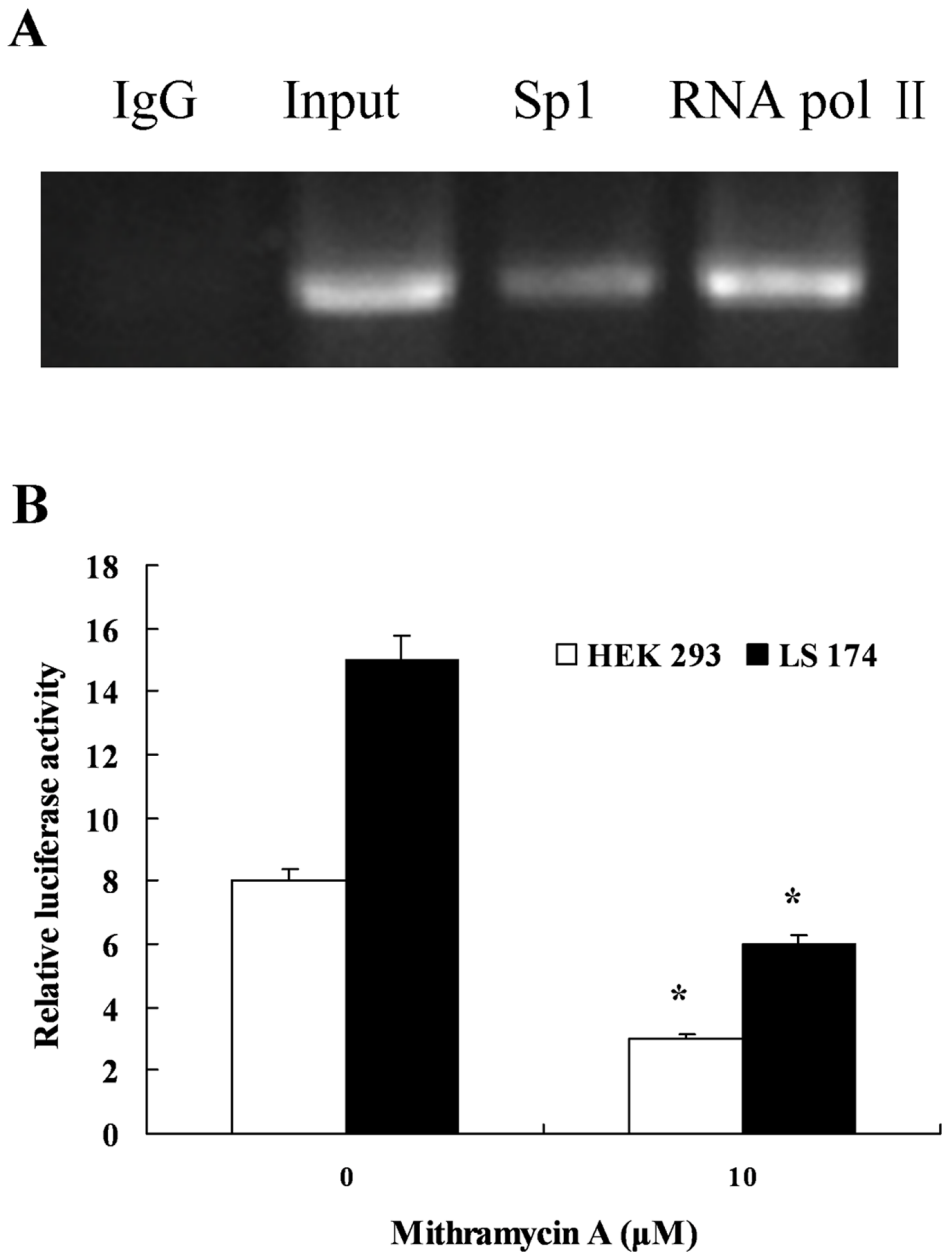
Verification of Sp1 binding at the *hTFF3* promoter. A. ChIP assay: LS174T cells were fixed with formalin, and then the chromatin was cleaved by sonication. The cleaved chromatin was then incubated with an anti-Sp1 antibody, a positive control RNA polII antibody, and a negative control IgG antibody. Finally, Sp1 binding at the *hTFF3* promoter was assessed by PCR amplification. B. HEK293 or LS174T cells were co-transfected with pGL3-300 and pRL-TK, and then treated with different concentrations of mithramycin A (a Sp1 inhibitor) for 24 h. Relative luciferase activity was measured and calculated. Data are presented as the mean ± SD (*P<0.05 *vs*. 0 µM mithramycin A).

We performed a ChIP assay to confirm the interactions between Sp1 and the *hTFF3* promoter. After fixation, ultrasonication, chromatin immunoprecipitation, de-cross-linking, and PCR, the DNA products were separated by gel electrophoresis. The results showed significant DNA bands in the Sp1-antibody immunoprecipitated samples and in the Input; however, no significant bands were observed in the negative control IgG samples (Fig. 4A).

Mithramycin A is a specific blocker of the Sp1 transcription factor, and it can specifically block Spl1-mediated transcriptional activity. HEK293 or LS174T cells were co-transfected with pGL3-300 and pRL-TK, and treated with different concentrations of mithramycin A for 24 h. The luciferase assay showed that the Sp1-specific blocker mithramycin A significantly inhibited the transcriptional activity of the *hTFF3* promoter (Fig. 4B).

### Transcriptional Activation of the *hTFF3* Promoter by Sp1

**Figure 5:**
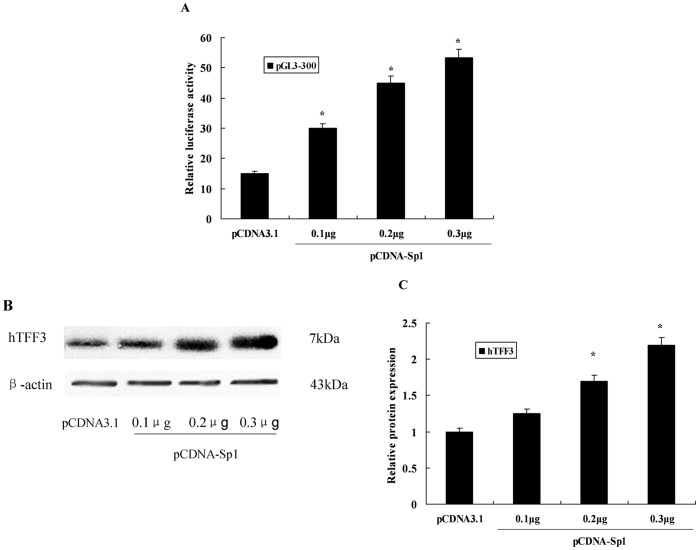
Effect of Sp1 overexpression on transcription from the *hTFF3* promoter. Both pGL3-300 and a Sp1 over-expression plasmid were co-transfected into LS-174T cells and cultured for 48 h. A. Relative luciferase activity was measured and calculated. Data are presented as the mean ± SD (*P<0.05 *vs*. PCDNA3.1). B and C. Detection of hTFF3 protein by western blotting (*P<0.05 *vs*. PCDNA3.1).

**Figure 6:**
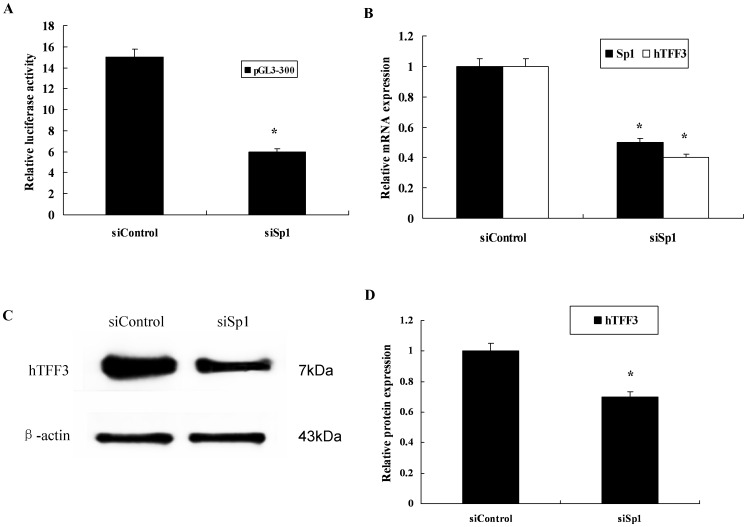
The effect of Sp1 down-regulation on transcription from the *hTFF3* promoter. pGL3-300, pRL-TK, and Sp1 siRNA or non-targeted control siRNA were co-transfected into LS-174T cells and cultured for 48 h. A. Relative luciferase activity (*P<0.05 *vs*. SiControl). B. Detection of *Sp1* and *hTFF3* mRNA levels by real-time PCR (*P<0.05 *vs*. SiControl). C and D. Detection of hTFF3 protein by western blotting.

To verify whether up-regulation of Sp1 affected the activity of the *hTFF3* promoter, cells were co-transfected with pGL3-300 and a Sp1 eukaryotic expression vector. We found that the higher the amount of the Sp1 vector, the higher the luciferase activity from the *hTFF3* promoter, indicative of dose-dependent activation (Fig. 5A). Western blot showed that the protein expression of hTFF3 was also significantly increased after Sp1 over-expression (Fig. 5B and 5C). We also down-regulated the expression of Sp1 using RNAi technology and observed the effect on the transcriptional activity of the *hTFF3* promoter. The results suggested that the activity of the *hTFF3* promoter was significantly decreased after treatment with Sp1-specific RNAi (Fig.6A). Real-time RT-PCR analysis showed that the mRNA expression of *Sp1* and *hTFF3* was significantly decreased after Sp1 RNAi treatment (Fig. 6B); Western blot results suggested that the protein expression of hTFF3 was also significantly decreased after RNAi (Fig. 6C and 6D).

## Conclusion

hTFF3 is a small peptide with potential medicinal value. Its main pharmacological functions are to alleviate gastrointestinal mucosal injuries caused by a variety of factors, and promote the repair of damaged mucosa [7], [8]. The hTFF3 cDNA is 222 bp in length (GenBank accession No. NM003226.3) and it encodes 73 amino acids, including 14 that encode forward signal peptide and 59 backward mature amino acids [9], [10]. A variety of substances have been found to regulate the expression of hTFF3, and its corresponding response elements, such as estrogen, upstream stimulatory factor (USF), interleukins, and hypoxia-inducible factor 1 (HIF-1), etc [11]. However, the core promoter sequence of *hTFF3* and the regulation of its transcription were not yet known. In this study, we cloned the region containing the human *hTFF3* gene promoter, determined the core promoter and the transcription factors that bind to the *hTFF3* gene promoter, and laid the foundation for the regulation of *hTFF3* expression. The luciferase reporter system used in this study was the first non-isotopic genetic reporting system widely used in mammals. pGL3-Basic contains a firefly luciferase reporter gene without a promoter. When the hTFF3 promoter was inserted into the multiple cloning sites of pGL3-Basic, this promoter promoted the transcription of the downstream firefly luciferase gene. The activity of the promoter is reflected by the firefly luciferase activity [12], [13]. As endogenous luciferase activity is barely detected in mammalian cells, there is no background signal. Therefore, firefly luciferase is an ideal reporter molecule in mammalian cells, and it has been widely used [14], [15]. In this study, we used the whole blood genome as a template to amplify the hTFF3 promoter sequences from −1,826 bp to +19 bp. This sequence was then inserted into the luciferase reporter vector pGL3-Basic. This region was shown to have strong promoter activity. Then, we detected significant changes in promoter activity when the 5′ end of the promoter fragment was moved from −300 bp to −200 bp. Therefore, we speculated that the core promoter of hTFF3 was included in this region (−300 bp to −200 bp). Four additional truncated vectors were constructed in this 100-bp region. We found that the activity of the construct that included the region from −280 bp to +19 bp was significantly decreased, indicating that the region from −300 bp to −280 bp was essential to maintain basic transcriptional activity. For this 20-bp region, we mutated five bases at a time to generate four consecutive mutants. The results showed that there were no changes in the activity of Mut-2, Mut-3, and Mut-4, which were close to that of pGL3-300. However, Mut-1 activity was significantly lower, indicating that there were response elements in the region from −300 bp to −296 bp that were needed to maintain basal transcriptional activity. Then, we used AliBaba 2.1 and the TRANSFAC database for bioinformatics analysis of the hTFF3 promoter. There was exactly one SP1 binding site (located from −301 bp to −293 bp) in Mut-1 mutation region. Therefore, we speculated that the region from −300 bp to −296 bp contained an SP1 binding site. In addition, we also found an interesting phenomenon, transient transfection of HEK293 cells and LS-174T cells with an *hTFF3* promoter plasmid showed that the activity of the *hTFF3* promoter was significantly higher in LS-174T cells than in HEK293 cells. LS-174T cells are derived from colon carcinoma, and have a goblet cell phenotype and mucus secretion characteristics. The cell specificity of the *hTFF3* promoter coincided with the characteristics of hTFF3-specific expression in the gastrointestinal tract. We predicted that there were Sp1 binding sites in the core region of the *hTFF3* promoter by site-directed mutagenesis combined with bioinformatic analysis. Then we confirmed the role of SP1 on hTFF3 transcription first by performing a ChIP experiment, the current best method for studying transcription factors at specific promoters *in vivo*, using the core *hTFF3* promoter (pGL3-300). The results suggested that Sp1 could bind to the core region of the *hTFF3* promoter *in vivo*. Then, we used a specific blocker of Sp1, mithramycin A, to inhibit the binding of Sp1 to the promoter. We treated cells with different concentrations of mithramycin A for 24 h. The results of the luciferase activity assay showed that mithramycin A could significantly inhibit the activity of the *hTFF3* promoter. These results confirmed that Sp1 could bind to the *hTFF3* promoter. The regulation of the *hTFF3* promoter was further confirmed by Sp1 over-expression and RNA interference assays. We first constructed a Sp1 eukaryotic expression vector, which was co-transfected with pGL3-300 into LS-174T cells. We found that cotransfection of the Sp1 expression vector and pGL3-300, significantly enhanced the activity of pGL3-300 in a dose-dependent manner. We also down-regulated the expression of Sp1 using RNAi technology and observed the changes in *hTFF3* promoter activity and Sp1 expression. The results showed that the transcriptional activity of the *hTFF3* promoter was significantly decreased after treatment with Sp1-specific RNAi to approximately 40% of that before interference. Real-time RT-PCR analysis showed that the expression of *Sp1* mRNA after RNAi treatment was approximately 50% of that before interference, and the expression of *hTFF3* mRNA was approximately 40% of that before interference. The western blot results suggested that the expression of hTFF3 protein was also significantly decreased after RNAi. In eukaryotes, the composition of the TATA box, initiator sequences, and CpG islands often have important functions at the promoter. In particular, the TATA box (T-A-T-A-A/T-A) is required for transcription of most genes, and is generally located upstream of the transcription start site (from −35 bp/−26 bp) [16]. The TATA box is typically the main recognition sequence for RNA polII at the transcription start site [17]. In addition, most genes also contain initiator sequences that can fulfill the role of the promoter [18], [19]. The consensus initiator sequence is 5′-C/T-C/T-A+1-N-T/A-C/T-C/T-C/T, and it is not as highly conserved as the TATA box [20]. A CATAAA box and a sequence of T-C-A+1-T-G-G-C-T were found in hTFF3 promoter (Fig.1). Our study failed to found the typical TATA box, initiator sequences and CpG island in hITF promoter by bioinformatics analysis; These defects in the *hTFF3* promoter might be compensated by other means, thus ensuring effective transcription. Sp1 is a sequence-specific DNA binding protein that is widely distributed in various tissues and cells [21]. Many housekeeping genes and tissue-specific genes are regulated by Sp1, especially genes transcribed by promoters without TATA boxes [22]. Sp1 can identify the GC/GT box and bind to 5′-G/AC/TC/TC/ACGCCC/TC/A-3′ and similar sequences that are often associated with basal transcription. It is involved in the transcriptional regulation of factors in various growth-related signaling pathways, including cell apoptosis, angiogenesis, and tumor growth. Our experiments showed that the basal transcription of *hTFF3* was precisely regulated by the binding of the transcription factor Sp1 to the core region of the promoter.

In conclusion, in this study, we successfully amplified the *hTFF3* promoter region and determined the sequence of the core promoter (from −300 bp to −280 bp), and showed that the essential region for maintenance of basal transcriptional activity was −300 bp to −296 bp. Bioinformatic analysis confirmed that this region contained a Sp1 binding site, and we further confirmed that Sp1 binds to the core region of the *hTFF3* promoter, and regulated the expression of hTFF3, laying the foundation for clarifying the regulation mechanism of *hTFF3* expression.
